# Identifying the novel key genes in renal cell carcinoma by bioinformatics analysis and cell experiments

**DOI:** 10.1186/s12935-020-01405-6

**Published:** 2020-07-21

**Authors:** Yeda Chen, Di Gu, Yaoan Wen, Shuxin Yang, Xiaolu Duan, Yongchang Lai, Jianan Yang, Daozhang Yuan, Aisha Khan, Wenqi Wu, Guohua Zeng

**Affiliations:** 1 Department of Urology, Minimally Invasive Surgery Center, The First Affiliated Hospital of Guangzhou Medical University, Guangdong Key Laboratory of Urology, Kangda Road 1#, Haizhu District, Guangzhou, 510230 Guangdong China; 2 Department of Urology, Affiliated Cancer Hospital and Institute of Guangzhou Medical University, Guangzhou, China; 3 Department of Family Medicine, Yunshan Medical Hospital, Shenzhen, China

## Abstract

**Background:**

Although major driver gene have been identified, the complex molecular heterogeneity of renal cell cancer (RCC) remains unclear. Therefore, more relevant genes need to be identified to explain the pathogenesis of renal cancer.

**Methods:**

Microarray datasets GSE781, GSE6344, GSE53000 and GSE68417 were downloaded from Gene Expression Omnibus (GEO) database. The differentially expressed genes (DEGs) were identified by employing GEO2R tool, and function enrichment analyses were performed by using DAVID. The protein-protein interaction network (PPI) was constructed and the module analysis was performed using STRING and Cytoscape. Survival analysis was performed using GEPIA. Differential expression was verified in Oncomine. Cell experiments (cell viability assays, transwell migration and invasion assays, wound healing assay, flow cytometry) were utilized to verify the roles of the hub genes on the proliferation of kidney cancer cells (A498 and OSRC-2 cell lines).

**Results:**

A total of 215 DEGs were identified from four datasets. Six hub gene (SUCLG1, PCK2, GLDC, SLC12A1, ATP1A1, PDHA1) were identified and the overall survival time of patients with RCC were significantly shorter. The expression levels of these six genes were significantly decreased in six RCC cell lines(A498, OSRC-2, 786- O, Caki-1, ACHN, 769-P) compared to 293t cell line. The expression level of both mRNA and protein of these genes were downregulated in RCC samples compared to those in paracancerous normal tissues. Cell viability assays showed that overexpressions of SUCLG1, PCK2, GLDC significantly decreased proliferation of RCC. Transwell migration, invasion, wound healing assay showed overexpression of three genes(SUCLG1, PCK2, GLDC) significantly inhibited the migration, invasion of RCC. Flow cytometry analysis showed that overexpression of three genes(SUCLG1, PCK2, GLDC) induced G1/S/G2 phase arrest of RCC cells.

**Conclusion:**

Based on our current findings, it is concluded that SUCLG1, PCK2, GLDC may serve as a potential prognostic marker of RCC.

## Background

Renal cell carcinoma (RCC) is a common malignant tumor of the urinary system [1] with the main treatment being surgical resection. The prognosis of metastatic renal cell carcinoma is poor because it is not sensitive to chemotherapy or other form of treatment and has a 5- year survival rate which is less than 5% [2]. Although many studies have shown that the most common causes of RCC are genetic factors, lifestyle, genetic mutations, cell damage, etc., the pathogenesis of renal cancer is extremely complicated, and the current research has not yet been fully elucidated. Therefore, potential markers for efficient diagnosis and treatment are urgently needed. Microarray technology has enabled us to explore genetic alterations in RCC and has proven to be a useful method for identifying new biomarkers in diseases.

There is an increasing evidence of the abnormal expression of genes being involved in the development and progression of RCC. It has been reported that Solute carrier family 12 member 1 (SLC12A1), Sodium/potassium-transporting ATPase subunit alpha-1 (ATP1A1) and Pyruvate dehydrogenase E1 component subunit alpha (PDHA1) are differentially expressed in renal cancer tissues. These are closely linked to the occurrence and development of renal cancer [3–5]. The Cancer Genome Atlas (TCGA) database found that Succinate CoA ligase subunit alpha (SUCLG1), Phosphoenolpyruvate carboxykinase (PCK2) and Glycine dehydrogenase (GLDC) were differentially expressed in renal cancer, however, there is no scientific research that showed whether they are the oncogenic molecules in renal cancer. Therefore, this project investigated these three molecules to clarify their mechanism of action in renal cancer.

So far, for RCC patients, no clear and effective molecular marker has been found for the diagnosis or treatment of RCC. Molecular markers help to improve early diagnosis, predict prognosis, and guide the development of treatment plans for patients with RCC. With the advent of the concept of individualized treatment of tumors and the advent of precision medicine, we should continue to explore new molecular markers or possible molecules of RCC Marker combinations, with a view to discovering molecular markers or combinations thereof that can accurately diagnose or predict the prognosis of RCC.

Over the past few decades, microarray technology and bioinformatics analysis have been widely used to screen for genetic changes and has helped to identify differentially expressed genes (DEGs) and functional pathways involved in carcinogenesis and progression of RCC. However, the false positive rate in independent microarray analysis makes it difficult to achieve reliable results. In our study, 4 mRNA microarray datasets from Gene Expression Omnibus (GEO) were downloaded and analyzed to obtain DEGs from renal cancerous and non-cancerous tissues. Subsequently, further explorations were performed for the better understanding of the molecular mechanisms of renal cancer development and progression.

In this study, we analyzed the databases of GEO, TCGA, GEPIA, Oncomine, and found that six genes (SUCLG1, PCK2, GLDC, SLC12A1, ATP1A1, and PDHA1), are related to the survival prognosis of patients with renal cancer. Furthermore, the functional verification of three genes (SUCLG1, PCK2 and GLDC) was performed in vitro experiments.

## Materials and methods

### Microarray data

GEO is a public functional genomics data repository of high throughout gene expression data, microarrays, and chips [6]. Four gene expression datasets GSE781 [7], GSE6344 [8], GSE53000 [9] and GSE68417 [10] were downloaded from GEO GPL96, GPL96, GPL6244, GPL6244 platforms respectively. The GSE781 dataset contained 10 RCC tissue samples and 24 non-cancerous samples. The GSE6344 included 20 RCC samples and 20 non-cancerous samples. The GSE53000 contained 53 RCC samples and 6 non-cancerous samples. The GSE68417 included 29 ccRCC samples and 20 non-cancerous samples.

### Identification of DEGs

The DEGs between ccRCC and non-cancerous samples were screened using GEO2R which is an interactive web tool that allows users to compare two or more datasets in a GEO series for identifying DEGs in experimental conditions. The adjusted P-values (adj. P) and Benjamini and Hochberg false discovery rate were applied to provide a balance between discovery of statistically significant genes and limitations of false-positives. Probe sets without corresponding gene symbols or genes with more than one probe set were removed or averaged, respectively. Adj. P-value < 0.01 and logFC (fold change) > 1 were considered statistically significant.

### KEGG and GO enrichment analyses of DEGs

The Database for Annotation, Visualization and Integrated Discovery (DAVID; http://david.ncifcrf.gov) (version 6.8) [11] is an online biological information database that integrates biological data. DAVID is an analytic tool that provides a comprehensive set of functional annotation information of genes and proteins to users for extracting biological information. KEGG is a database resource for understanding high-level functions and biological systems from large-scale molecular datasets generated by high-throughput experimental technologies [12]. GO is a common bioinformatics tool that annotates and analyzes biological processes of the genes [13]. In order to explain the function of DEGs, biological analyses were performed using DAVID online database and P < 0.05 was considered statistically significant.

### PPI network construction and module analysis

The PPI network was predicted and structured using Search Tool for the Retrieval of Interacting Genes (STRING: http://string-db.org) (version 11.0) [14] through online database. Analyzing the functional interactions among proteins may provide further understanding of the mechanisms of origin and development of diseases. In our study, PPI network of DEGs was constructed using STRING database, and an interaction with a combined score > 0.4 was considered statistically significant. Cytoscape (version 3.7.1) is an open source bioinformatics software platform for visualizing molecular interaction networks [15]. The plug-in Molecular Complex Detection (MCODE) (version 1.5.1) of Cytoscape is an APP for clustering a given network based on topology to find densely connected regions [16]. The PPI networks were structured using Cytoscape software and the most significant module in the PPI networks of DEGs was identified using MCODE app. The criteria for selection included: MCODE scores > 5, node score cut-off = 0.2, degree cut-off = 2, max depth = 100 and k-score = 2. Subsequently, the KEGG and GO analyses for these genes in the module were performed using DAVID.

### Hub genes selection and analysis

The hub genes were selected with betweenness degrees ≥ 100. The co-expression genes network was analyzed using cBioPortal online platform (http://www.cbioportal.org) [17, 18]. The biological process analysis of hub genes was performed and visualized employing Biological Networks Gene Oncology tool (BiNGO) (version 3.0.3) plugin of Cytoscape [19]. Hierarchical clustering of hub genes was analyzed using UCSC Cancer Genomics Browser (http://genome-cancer.ucsc.edu) [20]. The overall survival of hub genes was performed employing Kaplan–Meier curve in GEPIA (Gene Expression Profiling Interactive Analysis) [21]. The expression profiles of 6 hub gene (SUCLG1, GLDC, SLC12A1, PCK2, ATP1A1, PDHA1) were analyzed and displayed using UALCAN (http://ualcan.path.uab.edu/), and Oncomine (https://www.oncomine.org/) online database.

### Reagents

RPMI-1640 medium was purchased from Gibco Thermo Fisher Scientific, Inc. Fetal bovine serum (FBS) was purchased from Biological Industries. SUCLG1 (catalog no. 8072), PCK2 (catalog no. 6924), GLDC (catalog no. 12794) were primary antibodies bought from Cell Signalling Technology, Inc. The GAPDH antibody (catalog no. ab9485), secondary goat anti-rabbit antibody (catalog no. ab9485) were purchased from Santa Cruz Biotechnology, Inc.

### Cell culture and transfection

Human renal carcinoma cell lines OSRC-2 and A498, obtained from the Cell Bank of Chinese Academy of Sciences (Shanghai, China), were cultured in RPMI-1640 medium supplemented with 10% FBS, and incubated at 37 °C with 5% CO_2_. Cells in the logarithmic growth phase were used for subsequent experiments. OS-RC-2 and A498 cells were cultured in 6-well plates or 60 mm^2^ medium dishes and transfected with over expressed plasmids and negative control (NC) sequences that were purchased from Shanghai GenePharma Co., Ltd. (Shanghai, China). Transfection was conducted using Lipofectamine^®^ 3000 (Thermo Fisher Scientific, Inc.). The cells were harvested at 48 h after transfection for reverse transcription-quantitative polymerase chain reaction (RT-qPCR) analysis.

### Reverse transcription-quantitative (RT-q) PCR

Cell culture dishes were placed on ice, the culture medium was removed and cells were washed thrice with PBS. Total RNA was extracted using a Trizol^®^ kit (Fastgene, Inc.; Catalog no. 220010). The extracted RNA was reverse transcribed into cDNA using a PrimeScript^®^ RT Reagent kit with DNA Eraser (Takara Bio Inc.), as per the manufacturer’s instructions. The sequences of the primers used were showed in Additional file 1: Table S1.

### Western blotting

Protein samples were lysed at 4 °C using RIPA lysis buffer for 10 min. The samples were then centrifuged at 12,000×*g* for 20 min at 4 °C. The supernatants were obtained, and the protein concentration was quantified using the Coomassie Brilliant Blue method. The samples were mixed with 3× sample buffer solution and boiled for 5 min. Protein samples were loaded on a 10% SDS-gel and resolved using SDS-PAGE for 2 h. The resolved proteins were then transferred to a nitrocellulose membrane, which were blocked with 5% skimmed milk for 2 h at room temperature and then membrane was cut according to the molecular weight of the protein of interest that was based on a pre-stained protein ladder. Subsequently, the membranes were incubated overnight at 4 °C with the primary antibody. The following day, the membranes were washed four times with TBST buffer and incubated with the appropriate secondary antibody (1:10,000) for 2 h at room temperature. These membranes were again washed four times with TBST and signals were visualized using enhanced chemiluminescent reagent. Densitometry analysis was performed using Image J.

### Cell viability assays

To perform cell viability assays, cells were counted and plated in the well of 96-well plate (1000 cells per well) 24 h after transfection. At the cell culture duration of 0, 24, 48, 72, and 96 h(s), the cell viability was measured using Cell Counting Kit-8 (CCK8) Assay Kit (Dojindo, China) according to the manufacturer’s protocol.

### Cell proliferation evaluation

The cell proliferation was detected using the incorporation of 5-Ethynyl-2′-deoxyuridine (EdU) with the EdU Cell Proliferation Assay Kit (Ribobio, Guangzhou, China). The cells were further incubated with 50 µM EdU for 2 h before fixation, permeabilization and EdU staining, which were also performed according to the manufacturer’s protocol. The cell nuclei were stained with Hoechst at a concentration of 1 µg/mL for 30 min. The proportion of cells that incorporated EdU was determined using the fluorescence microscopy.

### Transwell migration and invasion assays

Cells were trypsinized and the samples were centrifuged at 900× g for 3 min at room temperature. After discarding the supernatant, the samples were resuspended in RPMI-1640 medium, centrifuged at 900× g for 3 min at room temperature and the cells were washed with PBS. The cells were resuspended in 500 µl RPMI-1640 medium and the density of cells was determined by hemocytometer. For the invasion assays, Transwell membranes were coated with Matrigel. A total of 2 × 10^4^ cells were placed in the upper chamber of an 8-µm pores microporous Transwell insert and in the lower chamber, 500 µl RPMI-1640 supplemented with 10% FBS was added and then the cells were incubated. Migration and invasion was determined by counting the number of cells that had successfully migrated through the membrane (migration) or invaded through the Matrigel matrix (invasion). After 24 h, the chamber was removed, cells which had not migrated or invaded were removed using a cotton swab, and the insert was dried at room temperature. Cells were subsequently fixed with 4% paraformaldehyde for 15 min at room temperature and dyed for 5 min using the crystal violet. After the filter membrane had dried, the sample was photographed.

### Wound healing assay

The cells were first selected, digested and counted, then inoculated into 6-well plates and incubated overnight. The next day, SUCLG1, PCK2, GLDC and NC over expression plasmid were used for transfection. After 48 h of transfection and the confluence reaching to almost 100%, a monolayer of the cells was scratched with a 200 µl pipette tip and photographed using an inverted microscope at ×200. The 6-well plate was placed in the incubator and was again photographed after 24 h. The scratched areas in the two photos were compared.

### Flow cytometry

The effect of SUCLG1, PCK2, GLDC and NC over expression plasmid on cell cycle was detected. The cells to be treated were digested with trypsin, centrifuged at 900× *g* for 3 min at room temperature, then washed in PBS and suspended in 500 µl buffer solution and fixed with 70% alcohol for 2 h. Subsequently, 50 µl RNase A was added to the 450-µl Propidium Iodide. After fully mixing and incubating at room temperature for 5 min, the cells were washed with 500 µl buffer solution and resuspended. Proceeding further, 500 µl mixed propidium iodide was added for 30 min at 37 °C. The samples were analysed using flow cytometer (BD FACSCalibur).

### Statistical analysis

All data were analyzed using GraphPad Prism 7.0 statistical software (GraphPad Software, Inc., La Jolla, CA, USA) and SPSS 23.0 (IBM Corp., Armonk, NY, USA). The two-tailed paired Student’s t-test was conducted for the analysis of the two groups. One-way analysis of variance (ANOVA) test and Bonferroni post hoc test were used for evaluating the differences among the multiple groups and *P *< 0.05 was considered to indicate a statistically significant difference.

## Results

### Identification of DEGs in RCC

**Fig. 1:**
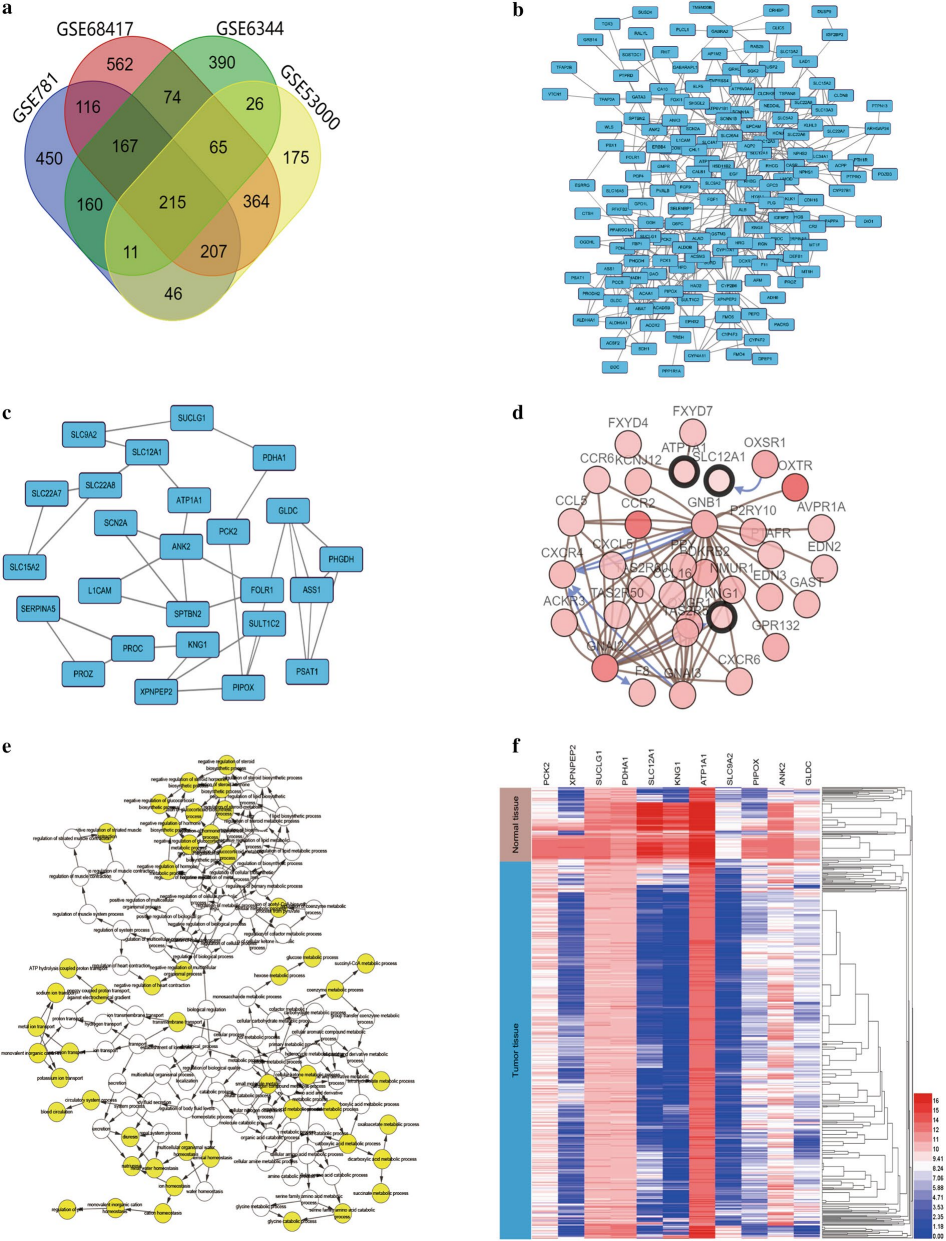
Identification of consistent DEGs in RCC. **a** Venn plot for consistent DEGs among the mRNA expression profiling sets GSE781, GSE68417, GSE6344, GSE53000. **b** The PPI network of DEGs was constructed using Cytoscape. **c** The most significant module was obtained from PPI network. **d** Hub genes and their co-expression genes were analyzed using cBioPortal. **e** The biological process analysis of hub genes was constructed. **f** Hierarchical clustering of hub genes was constructed using UCSC

After standardization of the microarray results, DEGs (1657 in GSE781, 1347 in GSE6344, 1161 in GSE53000 and 1807 in GSE68417) were identified between renal cancerous and non-cancerous tissues. The overlap among the 4 datasets included 215 genes as shown in the Venn diagram (Fig. 1a).

### KEGG and GO enrichment analyses of DEGs

To analyze the biological classification of DEGs, function and pathway enrichment analyses were performed using DAVID. GO analysis results showed that changes in biological processes (BP) of DEGs were significantly enriched in carboxylic acid, organic acid and oxoacid metabolic processes. Changes in cell component (CC) of DEGs were mainly enriched in extracellular exosome, vesicle and organelle. Changes in molecular function (MF) were mainly enriched in cofactor binding, coenzyme binding and secondary active transmembrane transporter activity. KEGG pathway analysis revealed that DEGs were mainly enriched in metabolic pathways, biosynthesis of antibiotics and Carbon metabolism (Table 1).

**Table 1:** GO and KEGG pathway enrichment analysis of DEGs in KIRC samples

Term/pathway ID	Description	Count in gene set	P-value
BP			
GO:0019752	Carboxylic acid metabolic process	45	1.07E−16
GO:0006082	Organic acid metabolic process	47	1.23E−16
GO:0043436	Oxoacid metabolic process	45	1.33E−16
GO:0044282	Small molecule catabolic process	25	6.84E−12
GO:0001822	Kidney development	22	1.91E−11
GO:0072001	Renal system development	22	5.72E−11
GO:0055114	Oxidation–reduction process	40	8.91E−11
GO:0016054	Organic acid catabolic process	19	3.42E−10
GO:0001655	Urogenital system development	22	5.53E−10
GO:0006811	Ion transport	48	8.49E−10
CC			
GO:0070062	Extracellular exosome	101	9.00E−25
GO:1903561	Extracellular vesicle	101	1.34E−24
GO:0043230	Extracellular organelle	101	1.38E−24
GO:0031988	Membrane-bounded vesicle	107	7.12E−20
GO:0044421	Extracellular region part	108	5.23E−18
GO:0005576	Extracellular region	113	1.48E−14
GO:0045177	Apical part of cell	26	1.04E−11
GO:0016323	Basolateral plasma membrane	20	2.35E−11
GO:0016324	Apical plasma membrane	23	4.15E−11
GO:0098590	Plasma membrane region	39	3.45E−10
MF			
GO:0048037	Cofactor binding	20	2.23E−09
GO:0050662	Coenzyme binding	14	7.50E−07
GO:0015291	Secondary active transmembrane transporter activity	15	1.92E−06
GO:0008509	Anion transmembrane transporter activity	14	2.26E−06
GO:0022804	Active transmembrane transporter activity	18	5.05E−06
GO:0016614	Oxidoreductase activity, acting on CH–OH group of donors	11	1.05E−05
GO:1901681	Sulfur compound binding	13	4.69E−05
GO:0015103	Inorganic anion transmembrane transporter activity	10	5.01E−05
GO:0016709	Oxidoreductase activity, with incorporation or reduction of molecular oxygen	6	2.11E−04
GO:0042802	Identical protein binding	34	2.45E−04
KEGG			
hsa01100	Metabolic pathways	48	3.79E−09
hsa01130	Biosynthesis of antibiotics	18	6.50E−08
hsa01200	Carbon metabolism	12	2.45E−06
hsa04960	Aldosterone-regulated sodium reabsorption	6	4.38E−04
hsa00010	Glycolysis/Gluconeogenesis	7	8.33E−04
hsa00280	Valine, leucine and isoleucine degradation	6	1.05E−03
hsa00020	Citrate cycle (TCA cycle)	5	1.45E−03
hsa00260	Glycine, serine and threonine metabolism	5	3.88E−03
hsa04610	Complement and coagulation cascades	6	5.74E−03
hsa04966	Collecting duct acid secretion	4	9.94E−03

BP, biological process; CC, cellular component; MF, molecular function

### PPI network construction and module analysis

The PPI network of DEGs was constructed (Fig. 1b) and the most significant module was identified using Cytoscape (Fig. 1c). The functional analysis of genes contained in this module was done using DAVID. GO analysis results showed that the genes in this module were mainly enriched in potassium ion transport, cellular chemical homeostasis, coenzyme metabolic process in BP; in mitochondrial matrix, apical plasma membrane, extracellular exosome in CC; in active transmembrane transporter activity in MF; in citrate cycle, biosynthesis of antibiotics and carbon metabolism in KEGG (Table 2).

**Table 2:** GO and KEGG pathway enrichment analysis of DEGs in the most significant module

Term/Pathway ID	Description	Count in gene set	P-value
BP			
GO:0006813	Potassium ion transport	4	2.29E−04
GO:0055082	Cellular chemical homeostasis	5	5.69E−04
GO:0006732	Coenzyme metabolic process	4	7.29E−04
GO:0019752	Carboxylic acid metabolic process	5	9.93E−04
GO:0043436	Oxoacid metabolic process	5	1.02E−03
GO:0019725	Cellular homeostasis	5	1.02E−03
GO:0051186	Cofactor metabolic process	4	1.30E−03
GO:0006082	Organic acid metabolic process	5	1.41E−03
GO:0035383	Thioester metabolic process	3	1.44E−03
GO:0006637	Acyl-CoA metabolic process	3	1.44E−03
CC			
GO:0005759	Mitochondrial matrix	4	1.83E−03
GO:0016324	Apical plasma membrane	3	1.33E−02
GO:0070062	Extracellular exosome	6	1.70E−02
GO:1903561	Extracellular vesicle	6	1.74E−02
GO:0043230	Extracellular organelle	6	1.74E−02
GO:0044429	Mitochondrial part	4	1.94E−02
GO:0045177	Apical part of cell	3	1.98E−02
GO:0030315	T-tubule	2	2.63E−02
GO:0014704	Intercalated disc	2	3.06E−02
GO:0044291	Cell–cell contact zone	2	4.08E−02
MF			
GO:0022804	Active transmembrane transporter activity	3	2.22E−02
KEGG			
hsa00020	Citrate cycle (TCA cycle)	3	5.07E−04
hsa01130	Biosynthesis of antibiotics	4	1.44E−03
hsa01200	Carbon metabolism	3	7.02E−03
hsa04964	Proximal tubule bicarbonate reclamation	2	2.65E−02
hsa01100	Metabolic pathways	5	3.76E−02
hsa00260	Glycine, serine and threonine metabolism	2	4.45E−02
hsa00620	Pyruvate metabolism	2	4.56E−02

BP, biological process; CC, cellular component; MF, molecular function

### Hub gene selection and analysis

**Fig. 2:**
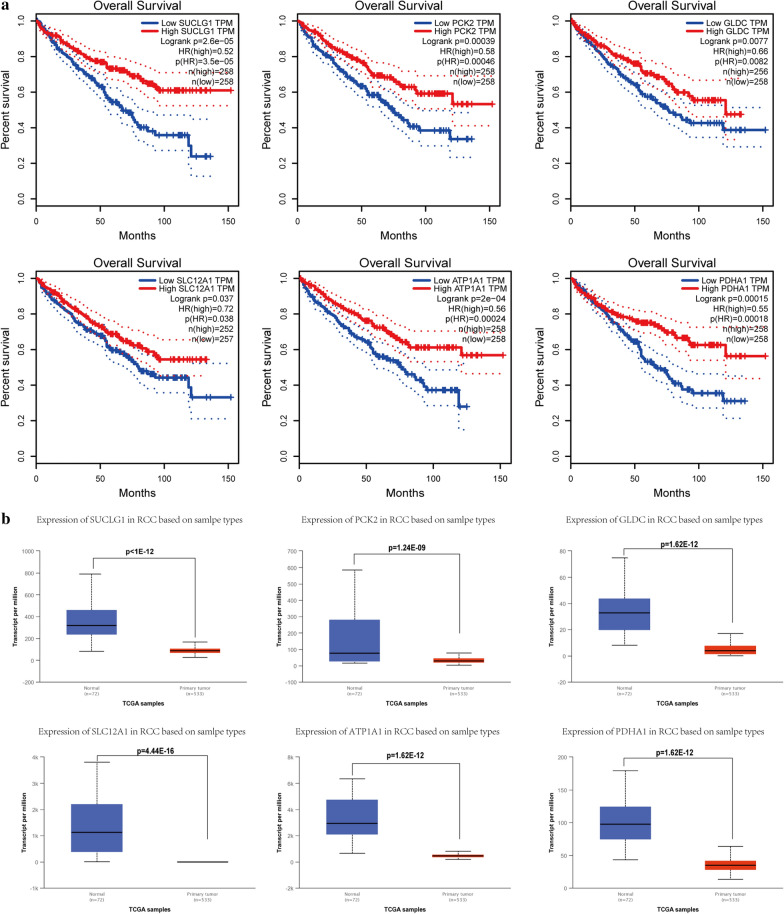
Survival and different analysis of the hub genes. **a** The survival curve of SUCLG1, PCK2, GLDC, SLC12A1, ATP1A1, PDHA1. **b** Analysis of differential expression in normal controls and patients with cancer based on SUCLG1, PCK2, GLDC, SLC12A1, ATP1A1, PDHA1 using the sample of The Cancer Genome Atlas database

A total of 11 genes were identified as hub genes with betweenness degrees ≥ 100. The hub gene symbol, abbreviations and functions are shown in (Table 3). A network of the 11 hub genes and their co-expression genes was showed using cBioPortal online platform (Fig. 1d). The biological process analysis of the hub genes is shown in Fig. 1e. Hierarchical clustering showed that hub genes could basically differentiate the kidney cancer samples from the noncancerous samples (Fig. 1f). Subsequently, the overall survival analysis of 11 hub genes was performed using Kaplan–Meier curve in the GEPIA dataset. Kidney cancer patient with SUCLG1, GLDC, SLC12A1, PCK2, ATP1A1 and PDHA1 alteration showed highest mortality (Fig. 2a). The expression profile of the 6 genes in human tissue was displayed using UALCAN (Fig. 2b) and Oncomine (Additional file 2: Fig).

**Table 3:** Functional roles of 11 hub genes with betweenness degrees ≥ 10

No.	Gene symbol	Full name	Function
1	ANK2	Ankyrin 2	Attaches integral membrane proteins to cytoskeletal elements.Also binds to cytoskeletal proteins. Plays a role in endocytosis and intracellular protein transport
2	SUCLG1	Succinate-CoA Ligase Alpha Subunit	Succinyl-CoA synthetase functions in the citric acid cycle, coupling the hydrolysis of succinyl-CoA to the synthesis of either ATP or GTP
3	GLDC	Glycine Decarboxylase	The P protein (GLDC) binds the alpha-amino group of glycine through its pyridoxal phosphate cofactor; CO_2_ is released and the remaining methylamine moiety is then transferred to the lipoamide cofactor of the H protein (GCSH)
4	SLC12A1	Solute Carrier Family 12 Member 1	Mediates sodium and chloride reabsorption. Plays a vital role in the regulation of ionic balance and cell volume
5	PIPOX	Pipecolic Acid And Sarcosine Oxidase	Metabolizes sarcosine, L-pipecolic acid and l-proline
6	KNG1	Kininogen 1	Kininogens 1 is inhibitors of thiol proteases; plays an important role in blood coagulation; inhibits the thrombin- and plasmin-induced aggregation of thrombocytes
7	SLC9A2	Solute Carrier Family 9 Member A2	Involved in pH regulation to eliminate acids generated by active metabolism or to counter adverse environmental conditions
8	PCK2	Phosphoenolpyruvate Carboxykinase 2	Catalyzes the conversion of oxaloacetate to phosphoenolpyruvate
9	XPNPEP2	X-Prolyl Aminopeptidase 2	Membrane-bound metalloprotease which catalyzes the removal of a penultimate prolyl residue from the N-termini of peptides. May play a role in the metabolism of the vasodilator bradykinin
10	ATP1A1	ATPase Na^+^/K^+^ Transporting Subunit Alpha 1	This action creates the electrochemical gradient of sodium and potassium ions, providing the energy for active transport of various nutrients
11	PDHA1	Pyruvate Dehydrogenase E1 Alpha 1 Subunit	The pyruvate dehydrogenase complex catalyzes the overall conversion of pyruvate to acetyl-CoA and CO2, and thereby links the glycolytic pathway to the tricarboxylic cycle.

### Expression level of key genes in human RCC cell lines and tissues

**Fig. 3:**
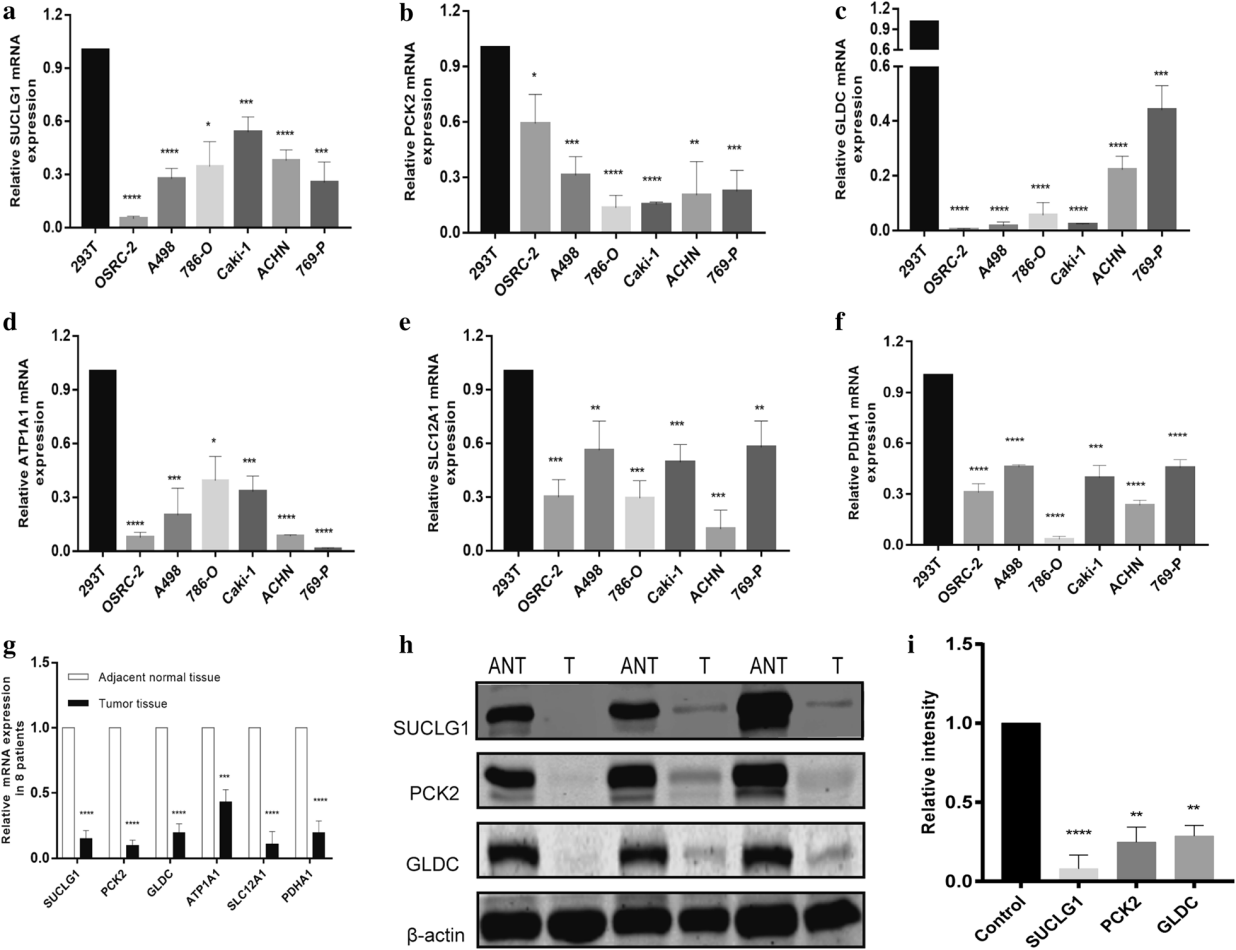
RT-qPCR and western blotting of the expression level of key genes in RCC cell lines. **a–f** RT-qPCR detection of mRNA expression of SUCLG1, PCK2, GLDC, SLC12A1, ATP1A1, PDHA1 in seven RCC cell lines. **g** Key genes mRNA expression level in eight paired kidney tumor tissues. **h** SUCLG1, PCK2, GLDC protein expression level in eight paired kidney tumor tissues (T) and their adjacent normal tissues (ANT). **i** The values of the band intensity represent the densitometric estimation of each band normalised by β-actin in (H). (*p < 0.05, **p < 0.01, ***p < 0.001, and ****p < 0.001)

qRT-PCR analysis revealed that 6 genes (SUCLG1, PCK2, GLDC, SLC12A1, ATP1A1 and PDHA1) relative expression was significantly decreased in 6 RCC cell lines compared to 293t cell line (Fig. 3a–f). Their expressions were also detected in primary tumor tissue and normal tissues using RT-PCR and Western blots. As expected, the mRNA expression and protein levels of the genes were down regulated in RCC samples compared to those in paracancerous normal tissues (Fig. 3g–i).

### Over expression of the key genes inhibits the proliferation of RCC

**Fig. 4:**
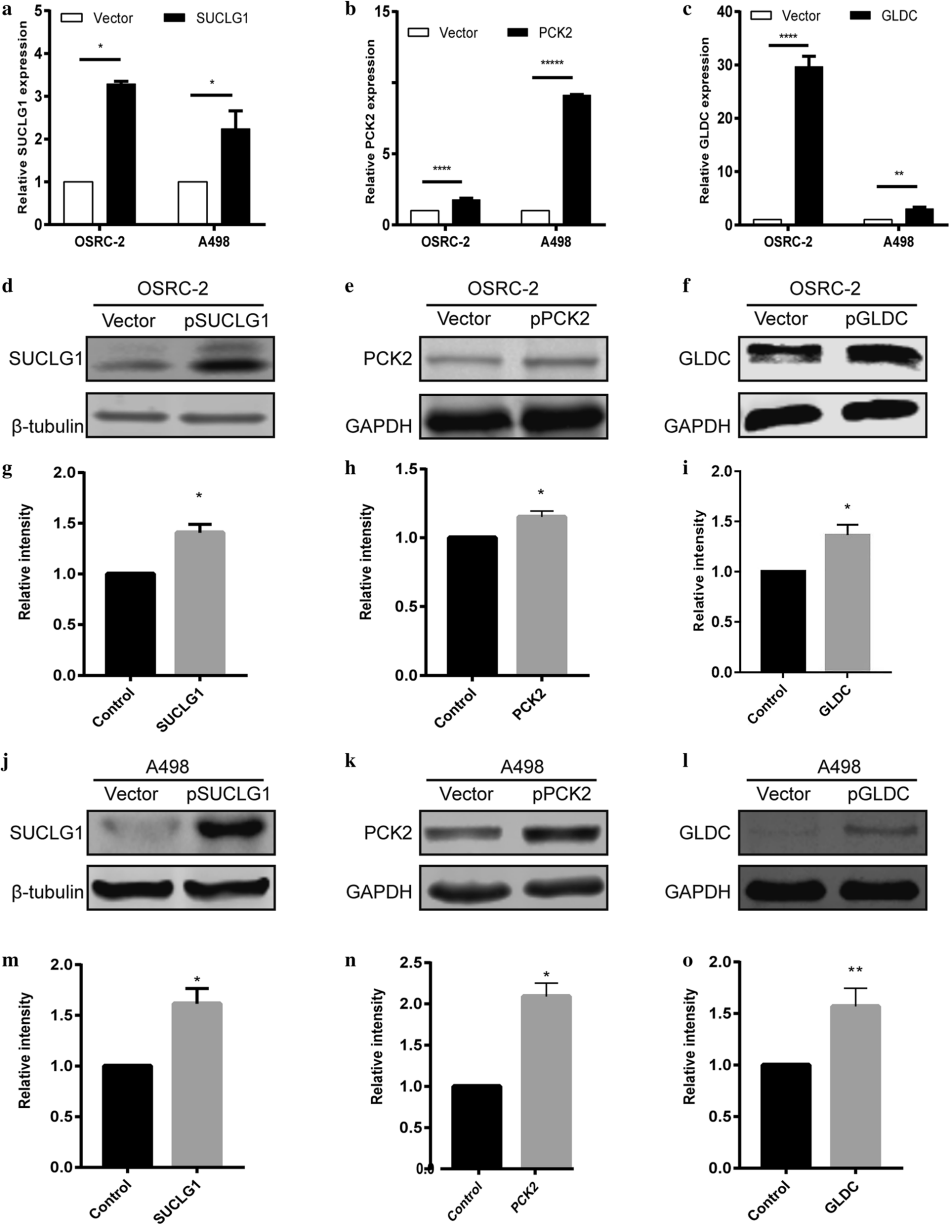
RT-qPCR and western blotting validation of the expression level of SUCLG1, PCK2, GLDC in OSRC-2 and A498 cell lines. **a–c** Forty-eight hours after plasmid or vector transfection, qPCR detected the expression level of 3 genes in both OSRC-2 and A498 cell lines. **b–f** Protein expression was evaluated by Western blot. **j–l** The values of the band intensity represent the densitometric estimation of each band normalised by β-actin in (**b–f**, respectively). (*p < 0.05 and **p < 0.01)

**Fig. 5:**
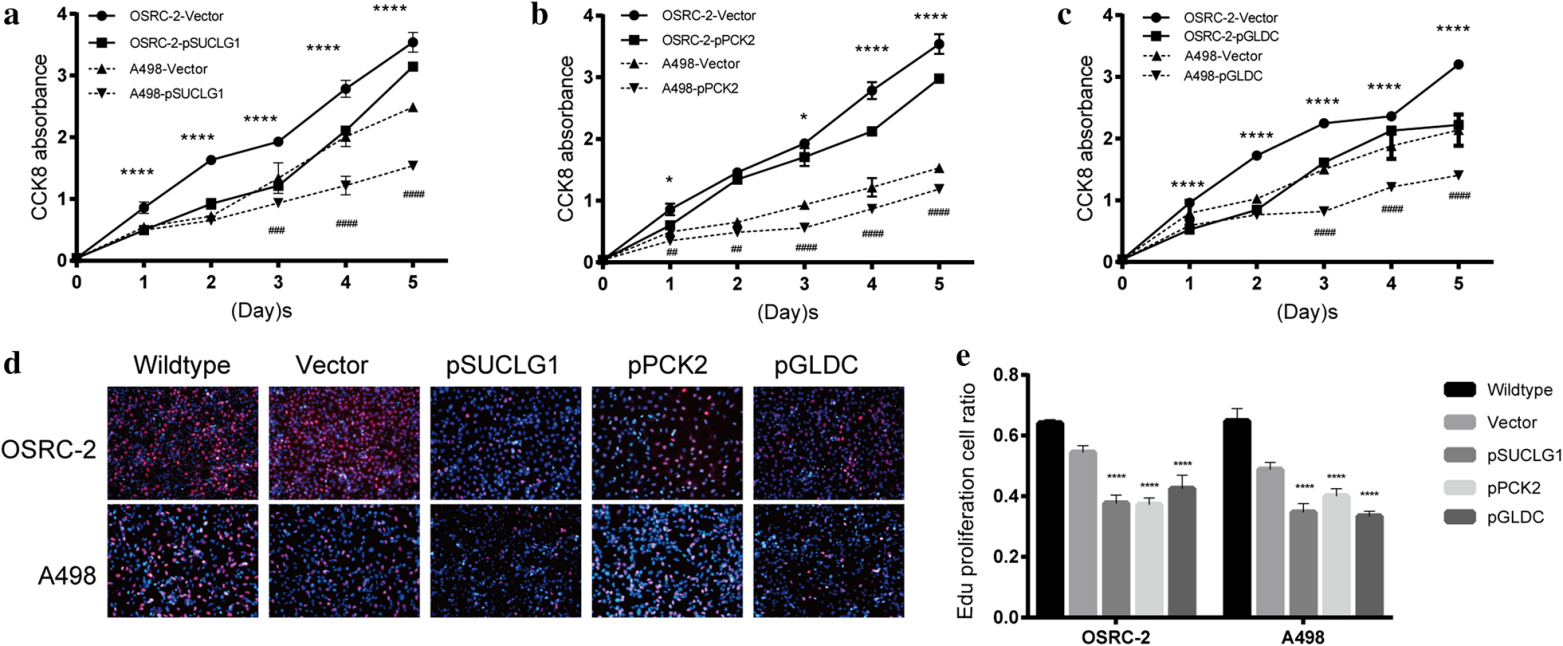
CCK-8 and Edu cell proliferation assay were performed after SUCLG1, PCK2, GLDC over-expression in OSRC-2 and A498. **a**–**c** CCK-8 cell proliferation assay. **d** Edu cell proliferation assay. **e** Quantification to Edu cell proliferation assay. (*p < 0.05, **p < 0.01, ***p < 0.001, and ****p < 0.001)

3 genes (SUCLG1, PCK2, GLDC) have not yet been researched to show the association with RCC, so they were selected as the main study molecules. In order to evaluate the potential role of the 3 genes in RCC cell proliferation, the cell lines that were linearly over expressed SUCLG1, PCK2, GLDC were established. Their protein expression levels (transfected and control cells) were determined by q-PCR and Western blotting, which showed an increase in expression of both OSRC-2 and A498 cells compared to the empty vector-transfected cells (Fig. 4). Cell viability was assessed by CCK-8 and Edu assay, and it was found that the number of OSRC-2 and A498 cells in which 3 genes were over expressed was decreased significantly. This illustrated that the 3 genes over expression reduced cellular growth and proliferation compared to the control cells (Fig. 5).

### SUCLG1, PCK2, GLDC inhibits the migration and invasion abilities of RCC cells

**Fig. 6:**
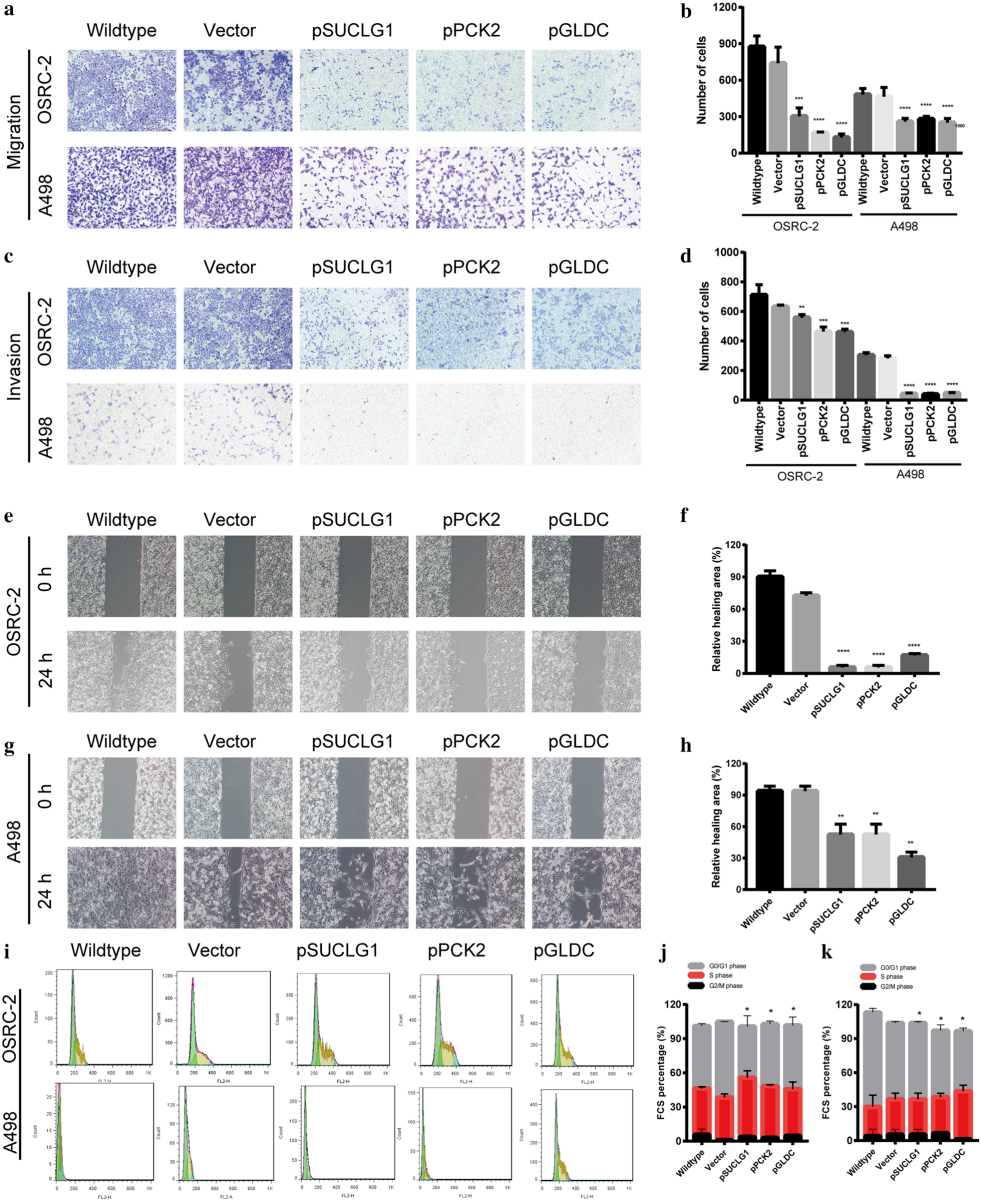
Over-expression of SUCLG1, PCK2, GLDC inhibits renal carcinoma cell migration and invasion in vitro. **a** Transwell cell migration assay was performed after the overexpression in OSRC-2 and A498 cells. **b** Quantitative analysis to (**a**). **c** Matrigel cell invasion assay was performed after the overexpression in OSRC-2 and A498 cells. **d** Quantitative analysis to (**c**). **e–g** Overexpression SUCLG1, PCK2, GLDC suppressed wound healing of OSRC-2 and A498 cell line. **f–h** Quantitative description to (**e**) and (**g**). **i** Cell cycle of overexpression SUCLG1, PCK2, GLDC after transfection 48 h was analyzed by flow cytometry. Image shows a representative experiment out of three. Data was performed as mean ± SD of three independent experiments. (*p < 0.05, **p < 0.01, ***p < 0.001, and ****p < 0.001)

To determine the effect of SUCLG1, PCK2, GLDC on the migration and invasiveness of RCC cells, transwell migration, matrigel invasion, and wound healing assays were performed. The number of migrated OSRC-2 cells in the experiment group was significantly decreased compared to the control group; the same result was observed in A498 cells of the experimental group (Fig. 6a, b). Additionally, 3 genes remarkably inhibited cell invasion abilities of OSRC-2 cells and A498 cells (Fig. 6c, d). Wound healing experiments revealed that the over expression SUCLG1, PCK2, GLDC significantly decreased the migration ability of OSRC-2 and A498 cells(Fig. 6e, h).

### SUCLG1, PCK2, GLDC induces G1/S/G2 phase arrest of RCC cells

To determine, whether or not the cell proliferation of RCC cells was associated with cell cycle arrest, we investigated the cell cycle of OSRC-2 and A498 cells. Flow cytometry data showed that the cycle progression of the two cell lines was obviously inhibited in G1/S/G2 phase. For SUCLG1and PCK2, the cell cycle was inhibited in S phase; For GLDC, G2/M phase was arrested (Fig.  6i, j).

## Discussion

In our present study, we investigated the roles of the SUCLG1, PCK2, GLDC in RCC. We found that the over expressions of these three genes significantly impaired the RCC process. Thus, targeting SUCLG1, PCK2, GLDC protein may be a useful strategy to inhibit tumor progression.

In the present study, four mRNA microarray datasets were analyzed to obtain DEGs between kidney cancer tissue and non-cancerous tissue. A total of 215 DEGs were identified in 4 datasets. GO and KEGG enrichment analysis indicated that these genes were mainly enriched in carboxylic acid, organic acid and oxoacid metabolic process, along with extracellular exosome, vesicle and organelle, including cofactor binding, coenzyme binding, secondary active transmembrane transporter activity, metabolic pathways, biosynthesis of antibiotics and carbon metabolism. Previous studies have reported that the dysregulation of carboxylic acid, organic acid and oxoacid play important role in the carcinogenesis or progression of cancers [22, 23]. Furthermore, it has been found that extracellular exosome, vesicle and organelle promote the tumor [24]. Moreover, studies have shown that cofactor binding, coenzyme binding, and secondary active transmembrane transporter activity often play a major role in the progression of cancer [25–27]. In conclusion, all these theories are consistent with our results. GO enrichment analysis revealed that changes in the most significant modules were mainly found in potassium ion transport, cellular chemical homeostasis, coenzyme metabolic process, mitochondrial matrix, apical plasma membrane, extracellular exosome and active transmembrane transporter activity, while changes in KEGG were mainly in citrate cycle, biosynthesis of antibiotics and carbon metabolism.

Using GEPIA for survival analysis of these 11 genes, showed that 6 genes (SUCLG1, PCK2, GLDC, SLC12A1, ATP1A1, PDHA1) had prognostic value in RCC patients, and may be involved in the carcinogenesis of the disease. Subsequently, We analyzed the expression of these 6 genes in 7 cell lines (293t, OSRC-2, A498, 786-O, Caki-1, ACHN, 769-P) and found that their expressions were all down-regulated. Meanwhile, tissues of 8 patients with renal cancer were randomly selected, and the expression of these 6 genes was detected in these tissues. The results showed that their expressions were also down-regulated. Furthermore, we tested the tissue samples of patients by western blot, suggesting that the protein expression was down-regulated (normal tissue adjacent to cancer vs tumor tissue). Many studies have shown that SLC12A1, ATP1A1, and PDHA1 are low-expressed in renal cancer and play an important role in the development of tumors. We have performed qPCR in cell lines and tissue of patients and the results are consistent with those of the predecessors. In order to clarify the molecular mechanism of these genes, we selected 3 molecules (SUCLG1, PCK2, GLDC) that have not previously been studied in renal cancer, and conducted in vitro experiments on them.

SUCLG1 is a rate-limiting enzyme for the formation of high-energy phosphate bonds between ATP and GTP in the tricarboxylic acid cycle, and its abnormal expression affects the energy metabolism, leading to tumorigenesis. Here, we showed that SUCLG1 was significantly down regulated in both human kidney cancer tissues and cell lines. PCK2 is a rate-limiting enzyme of the metabolic pathway and is involved in the gluconeogenesis pathway, tumorigenesis and development [28]. PCK2 expression is important in maintaining the tumor cells in vitro under limited glucose conditions and metabolic enzymes are necessary for proliferation and tumor growth in vivo. PCK2 has a tumor suppressing effect in melanoma and liver cancer [29, 30], and has a pro-cancer effect in lung and breast cancer [31, 32].

In melanoma, PCK2 down-regulation accelerates the biosynthesis and transport of citric acid from mitochondria to the cytoplasm. Over expression of PCK2 slows the growth of tumor repopulating cell (TRC) in vitro and reduces tumorigenesis in vivo [29]. In lung cancer samples, PCK2 activity is 3 times higher compared to the normal tissues [31]. In prostate cancer, PCK2 high-expressing patients have more aggressive tumors with low survival rates [28]. However, the function of PCK2 has not been investigated in kidney cancer. This experiment analyzed the expression of PCK2 in kidney cancer tissues and cell lines, and found that the expression of PCK2 is down-regulated. GLDC is mainly involved in regulating glycine metabolism and is one of the core metabolic enzymes for protein and amino acid metabolism. A research found that GLDC is over expressed in non-small cell lung cancer stem cells and accelerates the tumorigenesis by enhancing glycolysis and pyrimidine metabolism in non-cell lung cancer stem cells [33]. In addition, Jäger et al. found that GLDC was highly expressed in melanoma [34] whereas Min et al. found that GLDC was low expressed in gastric cancer [35]. Our study analyzed GLDC in renal cancer tissue and cells. The expression of GLDC was found to be down-regulated. In order to clarify the mechanisms of SUCLG1, PCK2, and GLDC in renal cancer, this study further focused on their proliferation, migration, invasion, and cell cycle of renal cancer cells through cell biology experiments.

Although the results of our current study indicated possible crucial roles of SUCLG1, PCK2, and GLDC in RCC, further studies, for example, the conduct of the rescue experiments, and in vivo studies will be helpful for the further validation of our present findings, and the better understanding of the mechanisms.

In conclusion, the experimental results showed that over expression of SUCLG1, PCK2, and GLDC can inhibit the progression of renal cancer cells. Therefore, UCLG1, PCK2, GLDC, have the potential to be a novel and valuable oncotarget protein for human kidney cancer.

## Supplementary information

**Additional file 1: Table S1.** The sequences of the primers

**Additional file 2: Figure S1.** Expression of genes in the Oncomine

## Data Availability

The datasets used and/or analyzed during the current study are available from the corresponding author on reasonable request

## Abbreviations

RCC: Renal cell carcinoma RCC
GEO: Gene Expression Omnibus
DEGs: Differentially expressed genes
PPI: Protein–protein interaction network
TCGA: The Cancer Genome Atlas
SLC12A1: Solute carrier family 12 member 1
ATP1A1: Sodium/potassium-transporting ATPase subunit alpha-1
PDHA1: Pyruvate dehydrogenase E1 component subunit alpha
SUCLG1: Succinate CoA ligase subunit alpha
PCK2: Phosphoenolpyruvate carboxykinase
GLDC: Glycine dehydrogenase
DAVID: Database for Annotation Visualization and Integrated Discovery
STRING: Search Tool for the Retrieval of Interacting Genes
MCODE: Molecular Complex Detection
BiNGO: Biological Networks Gene Oncology tool
GEPIA: Gene Expression Profiling Interactive Analysis
FBS: Fetal bovine serum
EdU: 5-Ethynyl-2′-deoxyuridin
ANOVA: One-way analysis of varianc
BP: Biological processes
CC: Cell component
MF: Molecular function
