# Correction: Identifying the novel key genes in renal cell carcinoma by bioinformatics analysis and cell experiments

**DOI:** 10.1186/s12935-022-02707-7

**Published:** 2022-10-06

**Authors:** Yeda Chen, Di Gu, Yaoan Wen, Shuxin Yang, Xiaolu Duan, Yongchang Lai, Jianan Yang, Daozhang Yuan, Aisha Khan, Wenqi Wu, Guohua Zeng

**Affiliations:** 1 Department of Urology, Minimally Invasive Surgery Center, Guangdong Key Laboratory of Urology, The First Affliated Hospital of Guangzhou Medical University, Kangda Road 1#, Haizhu District, Guangzhou, 510230 Guangdong China; 2 Department of Urology, Afliated Cancer Hospital and Institute of Guangzhou Medical University, Guangzhou, China; 3 Department of Family Medicine, Yunshan Medical Hospital, Shenzhen, China

## Correction to: Cancer Cell Int (2020) 20:331 10.1186/s12935-020-01405-6

In this article, the author would like to correct the duplication in Figs. 4 and 6 as mentioned below.

First, the Fig. 4g and Fig. 4i are the same. Figure 4g was copied to Fig. 4i by mistake, resulting in duplication of the two figures. Second, in Fig. 6, the wildtype and the vector of OSRC-2 were duplicated by mistake.

**Fig. 4:**
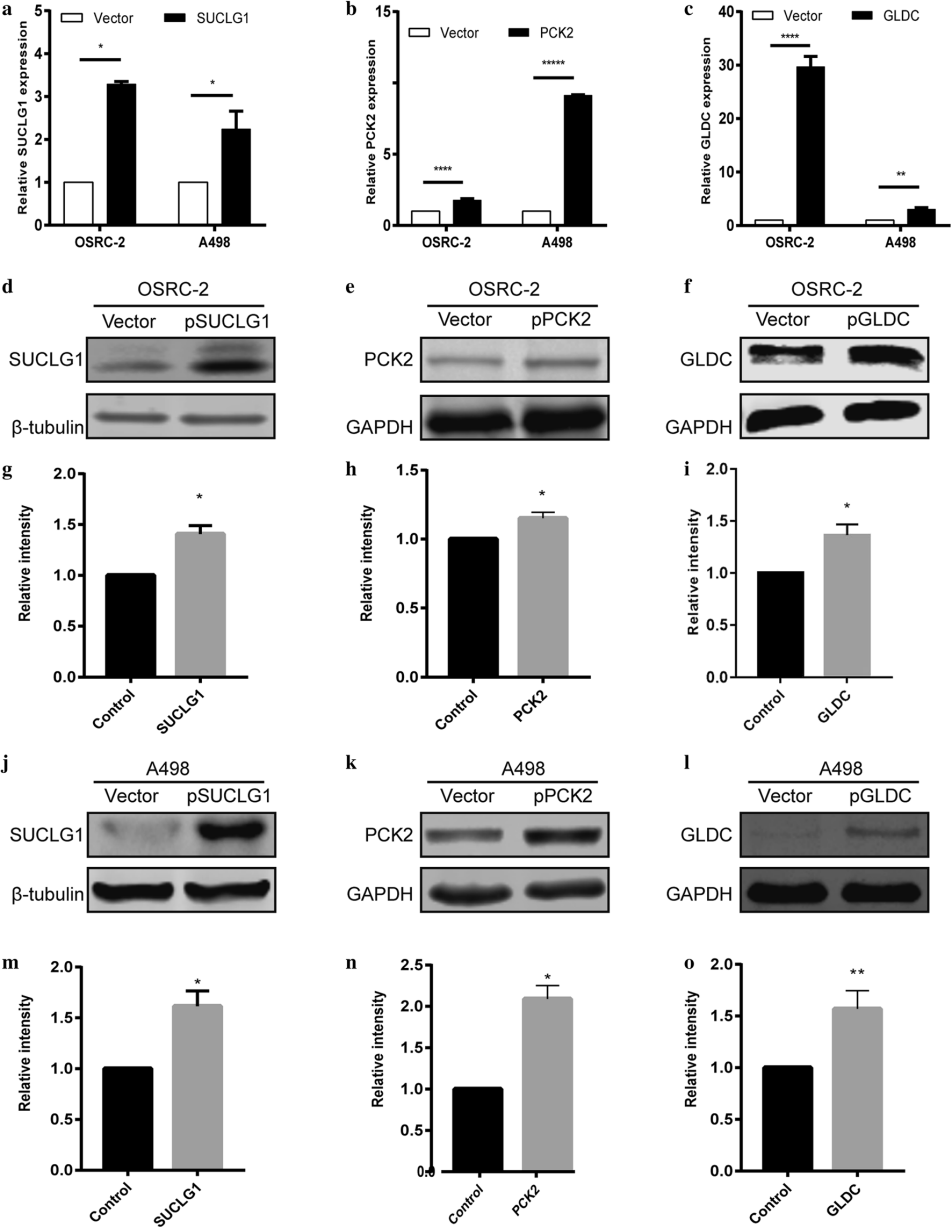
RT-qPCR and western blotting validation of the expression level of SUCLG1, PCK2, GLDC in OSRC-2 and A498 cell lines. **a**–**c** Forty-eight hours after plasmid or vector transfection, qPCR detected the expression level of 3 genes in both OSRC-2 and A498 cell lines. **b**–**f** Protein expression was evaluated by Western blot. **j**–**l** The values of the band intensity represent the densitometric estimation of each band normalised by β-actin in (**b**–**f**, respectively). (*p < 0.01)

**Fig. 6:**
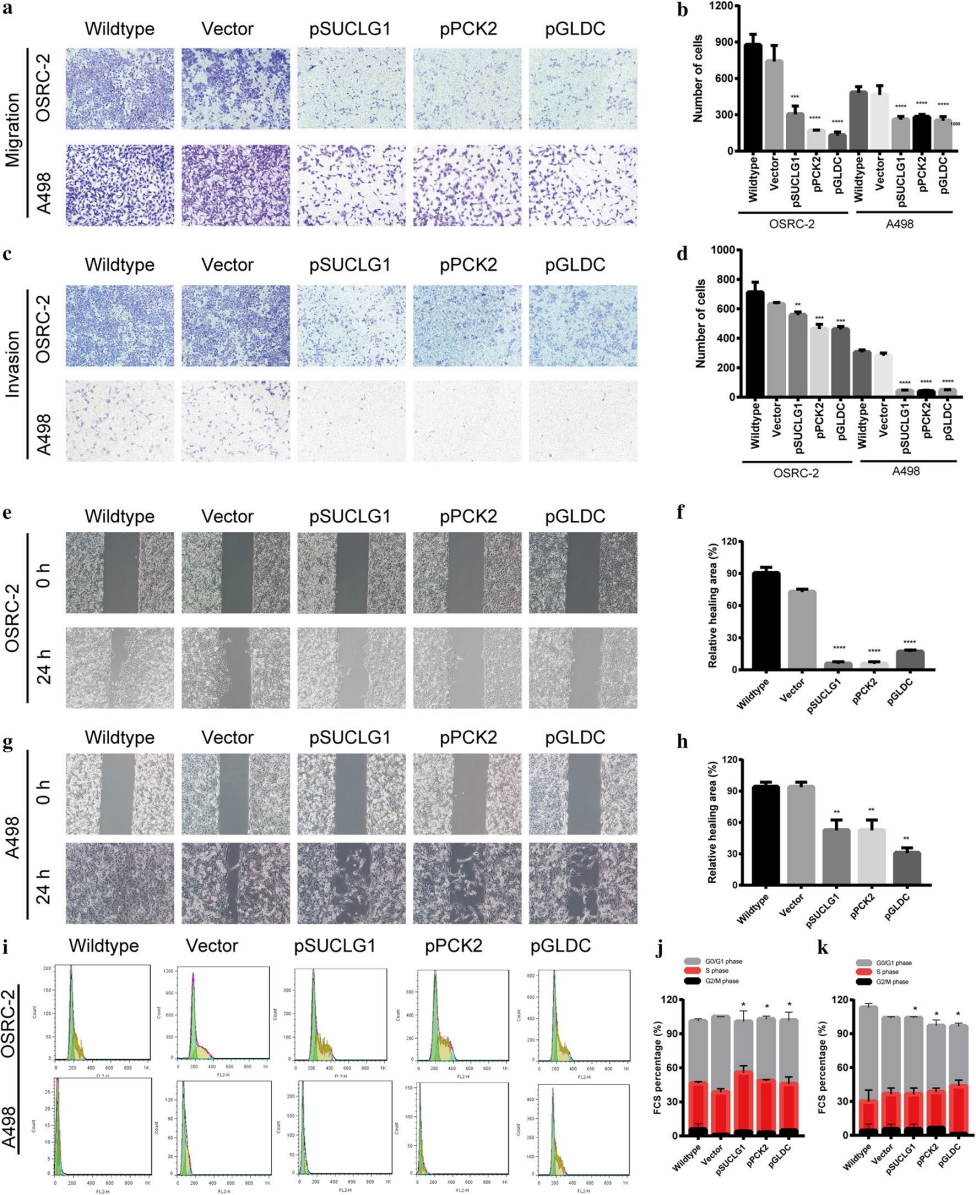
Over-expression of SUCLG1, PCK2, GLDC inhibits renal carcinoma cell migration and invasion in vitro. **a** Transwell cell migration assay was performed after the overexpression in OSRC-2 and A498 cells. **b** Quantitative analysis to (**a**). **c** Matrigel cell invasion assay was performed after the overexpression in OSRC-2 and A498 cells. **d** Quantitative analysis to (**c**). **e**–**g** Overexpression SUCLG1, PCK2, GLDC suppressed wound healing of OSRC-2 and A498 cell line. **f**–**h** Quantitative description to (**e**) and (**g**). **i** Cell cycle of overexpression SUCLG1, PCK2, GLDC after transfection 48 h was analyzed by fow cytometry. Image shows a representative experiment out of three. Data was performed as mean ± SD of three independent experiments. (*p < 0.001)

The correct Fig. 4i and correct graph of Vector of OSRC-2 are published with this correction.

The original article [1] has been corrected
