# Supplementary material for: Identifying the novel key genes in renal cell carcinoma by bioinformatics analysis and cell experiments
Source: Cancer Cell Int. 2020 Jul 21;20:331. doi: 10.1186/s12935-020-01405-6 (PMC7372855; doi:10.1186/s12935-020-01405-6)
Supplement: Supplementary file 1 — Additional file 1: Table S1. The sequences of the primers [file 12935_2020_1405_MOESM1_ESM.doc]

**Additional file 1: Table S1. The sequences of the primers**

| Gene | Primer | Base sequence |
| --- | --- | --- |
| SUCLG1 | forward | 5'-GAGCAACGGCTTCTGTCATTT-3' |
|  | reverse | 5'-TGCTTGACTCGTACCATGTCC-3' |
| PCK2 | forward | 5'-GCCATCATGCCGTAGCATC-3' |
|  | reverse | 5'-AGCCTCAGTTCCATCACAGAT-3' |
| GLDC | forward | 5'-CCAGACACGACGACTTCGC-3' |
|  | reverse | 5'-CAATTCATCAATGCTCGCCAG-3' |
| SLC12A1 | forward | 5'-AGTGCCCAGTAATACCAATCGC-3' |
|  | reverse | 5'-GCCTAAAGCTGATTCTGAGTCTT-3' |
| ATP1A1 | forward | 5'-ACAGACTTGAGCCGGGGATTA-3' |
|  | reverse | 5'-ACAGACTTGAGCCGGGGATTA-3' |
| PDHA1 | forward | 5'-TGGTAGCATCCCGTAATTTTGC-3' |
|  | reverse | 5'-ATTCGGCGTACAGTCTGCATC-3' |
