# Supplementary figures and images for: Identifying the novel key genes in renal cell carcinoma by bioinformatics analysis and cell experiments
Source: Cancer Cell Int. 2020 Jul 21;20:331. doi: 10.1186/s12935-020-01405-6 (PMC7372855; doi:10.1186/s12935-020-01405-6)

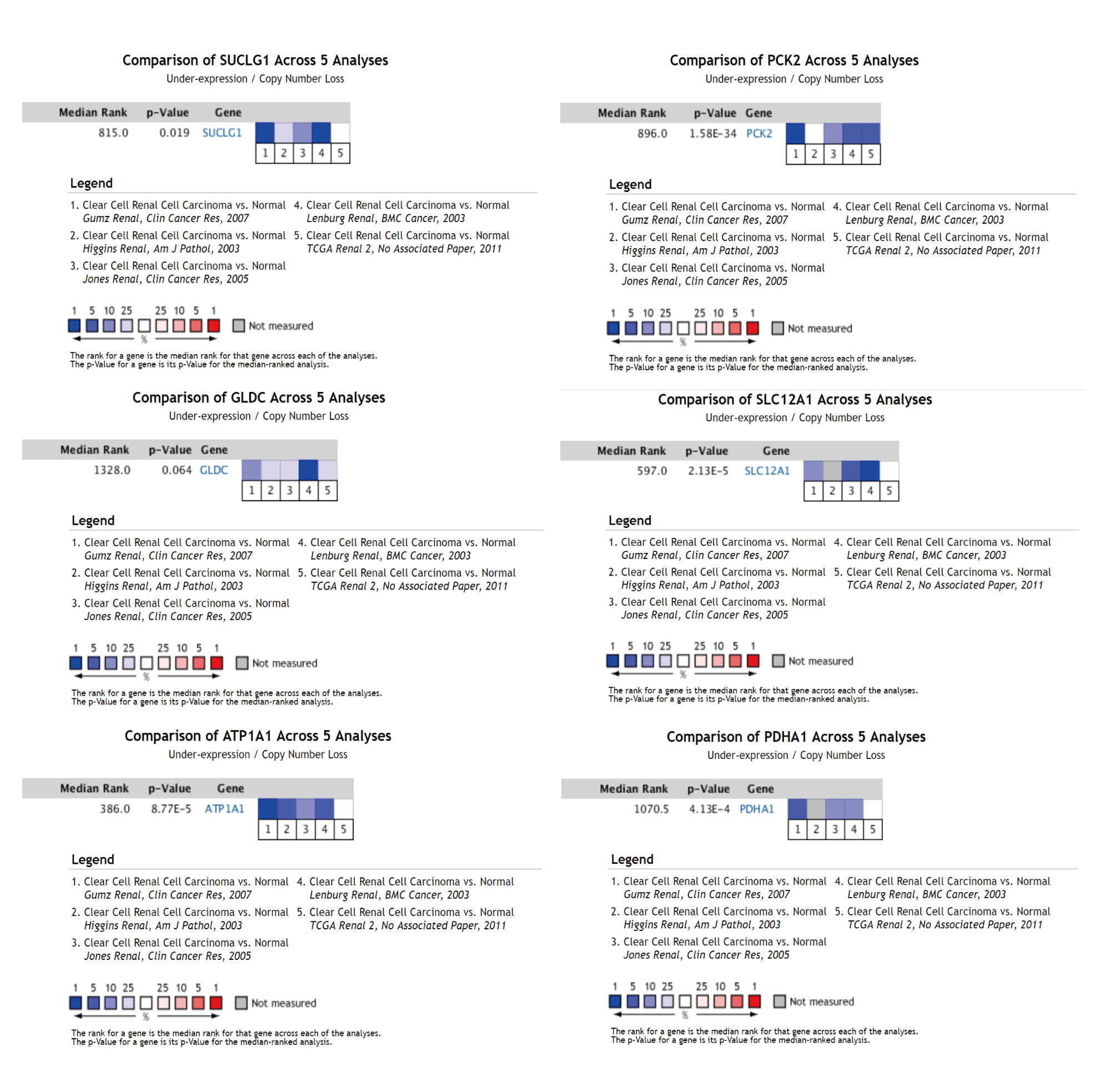

Supplement: Supplementary file 2 — Additional file 2: Figure S1. Expression of genes in the Oncomine [file 12935_2020_1405_MOESM2_ESM.tif]
